# Beneficial Effects of *Opuntia* spp. on Liver Health

**DOI:** 10.3390/antiox12061174

**Published:** 2023-05-29

**Authors:** Irene Besné-Eseverri, Jenifer Trepiana, Saioa Gómez-Zorita, Marilena Antunes-Ricardo, M. Pilar Cano, María P. Portillo

**Affiliations:** 1 Nutrition and Obesity Group, Department of Nutrition and Food Sciences, Faculty of Pharmacy, University of the Basque Country (UPV/EHU) and Lucio Lascaray Research Centre, 01006 Vitoria, Spain; irene.besne@ehu.eus (I.B.-E.); saioa.gomez@ehu.eus (S.G.-Z.); mariapuy.portillo@ehu.eus (M.P.P.); 2 CIBER Physiopathology of Obesity and Nutrition (CIBERobn), Institute of Health Carlos III, 01006 Vitoria, Spain; 3 BIOARABA Institute of Health, 01006 Vitoria-Gasteiz, Spain; 4 Tecnologico de Monterrey, Escuela de Ingeniería y Ciencias, Centro de Biotecnología-FEMSA, Av. Eugenio Garza Sada 2501 Sur, Monterrey 64849, Mexico; marilena.antunes@tec.mx; 5 Tecnologico de Monterrey, Institute for Obesity Research, Ave. Eugenio Garza Sada 2501 Sur, Monterrey 64849, Mexico; 6 Laboratory of Phytochemistry and Plant Food Functionality, Biotechnology and Food Microbiology Department, Institute of Food Science Research (CIAL) (CSIC-UAM), Nicolás Cabrera 9, 28049 Madrid, Spain; mpilar.cano@csic.es

**Keywords:** *Opuntia* spp., steatosis, steatohepatitis, liver damage, oxidative stress

## Abstract

The genus *Opuntia* spp. includes plants capable of growing in arid, temperate and tropical climates. The vast majority of wild species grow in Mexico, but *O. ficus-indica* (prickly pear or nopal) is cultivated around the world and it is one of the most studied. This review shows the currently available knowledge concerning the action of *O. ficus-indica* and other *Opuntia* species (*Opuntia vulgaris*, *Opuntia robusta*, *Opuntia streptacantha*, *Opuntia microdasys*, *Opuntia dillenii* and *Opuntia dejecta*) on liver health. The available data demonstrate the positive effects of extracts, vinegar, juices or seed oil of the *Opuntia* genus on the alterations induced in the liver by inadequate feeding patterns or the administration of chemicals. In this regard, the potential beneficial effects of nopal are related to the attenuation of triglyceride accumulation, oxidative stress and/or inflammation. Nevertheless, there is no information concerning the bioactive compound’s characterisation in most of these studies; consequently, it is not possible to link the therapeutic effects of these plants to the presence of specific compounds in the nopal extracts. Therefore, further research is needed to confirm if the positive effects observed in animal models are also found in humans, in order to determine whether *Opuntia* can represent an effective tool to prevent and/or manage hepatic alterations.

## 1. Introduction

Herbal and plant-based compounds represent a useful source of active substances, with beneficial roles for a great number of diseases. The use of these compounds predates contemporary medicine, and they were first used in ancient times. On the other hand, many existing medications are directly or indirectly derived from plants, and in recent years, interest in herbal and alternative medicines has been growing.

In this scenario, the genus *Opuntia* (*Cactaceae* family) includes different plants of great interest taking into account climate change because they are well adapted to arid and semi-arid zones. It encompasses plants of very different sizes, from the small *Opuntia microdasys* to shrubby or arboreal species with trunk and crown, such as *Opuntia leucotricha*, which can reach a height of 5 m. The segments (cladodes) are characteristic of the genus, with the appearance of a flat, fleshy and generally oval leaf. To date, more than 250 varieties of *Opuntia* spp. are known [1]. Although *Opuntia* spp. plants are native to Mexico and the highest wild species richness is found in this country [1,2], they are also located in different countries since they can grow in arid, temperate and tropical climates [3,4]. *Opuntia* products have great potential for the food, pharmaceutical and cosmetic industries and, thus, they are of great economic interest [5,6,7,8]. They contribute to the development of agriculture, especially in dry zones, since they need little water and are very productive, so they can be a good alternative for animal feed [5]. In addition, these plantations could protect the environment against erosion, allowing the fixation of the soil [9]. Diverse benefits of *Opuntia* spp. have been suggested by traditional medicine. Some of these beneficial effects have been analysed under a scientific basis [10]. As a result, it has been shown that *Opuntia* spp. extracts, obtained from cladodes, flowers or fruits, could be effective for the prevention or treatment of diabetes, obesity, non-alcoholic fatty liver disease and cardiovascular diseases and the proliferation of some cancer cell lines [1,5,6,7,11,12].

These effects are due, at least in part, to their anti-inflammatory and antioxidant properties [4,5,6,7,11,13], which are attributed to their rich content of bioactive compounds, phenolic compounds (flavonoids and phenolic acids), phytosterols, betalains and some carbohydrates [7,8,14,15,16,17]. The profile of bioactive compounds varies according to the species and the part of the plant [16]. It also depends on the cultivar, climatic conditions and the geographical area where the plant grows. Consequently, the biological properties of the *Opuntia* varieties can also differ [3,18]. Several published papers offer more precise information concerning the bioactive compound composition of different *Opuntia* varieties [3,13,14,18].

**Figure 1:**
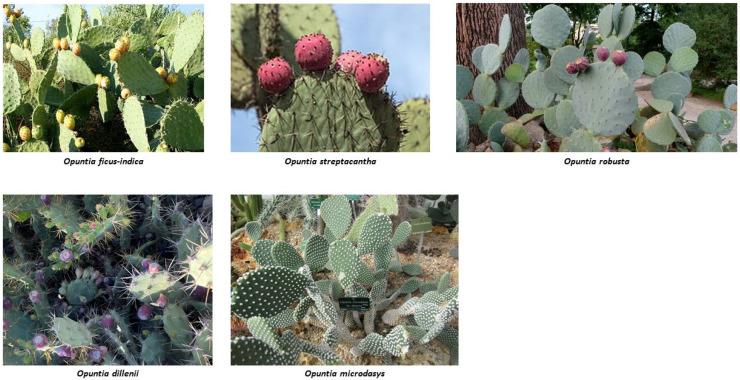
Different species of *Opuntia* spp.

This review aims to summarise the reported information regarding the beneficial effects of *Opuntia* spp. on liver steatosis and liver damage. The interest of these plants in liver steatosis comes from the fact that currently, there is no approved pharmacological treatment. Consequently, there is a growing interest in the development of new therapies. Although the number of reported studies is limited, the majority of them provide scientific evidence of its beneficial effects. It is important to point out that, to date, only pre-clinical studies carried out in rodent models, but not interventional studies in humans, have been published. Different species of *Opuntia* have been analysed, *Opuntia ficus-indica* being the most frequently studied (Figure 1). Moreover, different models of liver steatosis and damage have been studied.

## 2. Search Strategy

A bibliographic search was conducted to identify studies included in the PubMed medical database up to April 2023, using different combinations of the following keywords: steatosis, liver damage, hepatoprotective, *Opuntia* and cactus. Therefore, only original articles written in English were included. From the article collection obtained, 24 studies were preserved after screening the title, abstract and full text.

## 3. Effects of *Opuntia ficus-indica* on Liver Steatosis

*Opuntia ficus-indica* is the most extensively studied in scientific reports. Regarding the effects of this plant on liver steatosis, different animal models have been used (Table 1). Morán-Ramos et al. [19] carried out a study on Zucker (*fa*/*fa*) rats, a model of genetic obesity that exhibits fatty liver. The animals were divided into two groups and were fed either a control diet or a diet supplemented with a dehydrated extract, obtained from *Opuntia ficus-indica* cladodes, for seven weeks. *Opuntia ficus-indica* supplement was included in the diet in the amount needed to provide 4% of dietary fibre in lieu of the cellulose present in the control diet. No data were reported concerning the amount and composition of the bioactive compounds.

Liver weight, lipid accumulation in the hepatic intracellular vesicles, serum transaminases, alanine aminotransferase (ALT) and aspartate aminotransferase (AST) were lower in *Opuntia ficus-indica*-treated rats than in the controls. The study revealed various mechanisms of action underlying the anti-steatotic effect. On the one hand, *Opuntia ficus-indica* increased the gene expression of carnitine palmitoyltransferase-1 (*Cpt-1*) and acyl-CoA oxidase1 (*Aox*), two enzymes involved in fatty acid oxidation, including peroxisome proliferator-activated receptor α (*Ppar-α*), the transcription factor that regulates these enzymes, along with greater CPT-1 protein concentration. These results suggest that *Opuntia ficus-indica* increased the capacity of fatty acid oxidation, thus preventing their accumulation as triglycerides. By contrast, no changes were observed in several genes involved in *de novo* lipogenesis.

Taking into account that oxidative stress plays a key role in the etiopathogenia of liver steatosis, the antioxidant effect of *Opuntia* is of great interest in the prevention and treatment of fatty liver. Due to this fact, the authors also analysed the effect of *Opuntia ficus-indica* on several parameters related to oxidative stress, and they observed a reduction in the hepatic concentration of malondialdehyde (MDA), a biomarker of lipid peroxidation. Since no changes were observed in the activity of the antioxidant enzymes, glutathione peroxidase (GPx), superoxide dismutase (SOD) and catalase (CAT), nor in the expression of their genes, the authors concluded that the reduction in the oxidative stress induced by *Opuntia ficus-indica* was probably due to a direct interaction between antioxidant molecules present in this plant and the reactive species. Lastly, insulin signalling cascade function was improved by *Opuntia ficus-indica*, as shown by the reduced serum insulin level and the increase in insulin receptor substrate-1 (IRS-1) and protein kinase B (AKT) phosphorylation found in the rats treated with the extract.

Bouazza et al. [20] studied the effect of *Opuntia ficus-indica* in a dietary model of liver steatosis created by feeding rats a high-fat diet (HF). Thus, the authors assessed the antioxidant and hepatoprotective effects of the vinegar obtained from prickly pear (*Opuntia ficus-indica*) from Algeria in rats fed with an obesogenic diet. Rats supplemented with *Opuntia ficus-indica* received 7 mL of prickly pear vinegar/kg body weight (b.w.)/day, for seven months. The results demonstrated that the hepatic lipid profile was negatively affected as a consequence of HF feeding. Prickly pear vinegar treatment improved most of the analysed parameters, significantly reducing hepatic total lipids, total cholesterol and serum low-density lipoprotein-cholesterol (LDL-c) content and increasing serum high-density lipoprotein-cholesterol (HDL-c). In addition, HF augmented AST, ALT and alkaline phosphatase (ALP) levels, and although prickly pear vinegar administration decreased all parameters compared with the HF group, only AST reduction was significant.

Regarding the antioxidant parameters, HF reduced SOD, GPx, glutathione reductase (GRx) activities, total antioxidant status and copper, manganese and iron levels while augmenting the amount of hepatic thiobarbituric acid reactive substances (TBARS). However, prickly pear vinegar treatment was able to significantly prevent the changes observed in SOD and GPx activities, and a significant increase in copper and iron levels was also observed. In other respects, the histopathological study showed that HF caused the hepatocellular degeneration of fibrous tissues, vascular congestion, necrosis, the infiltration of the lymphocytes around the central vein and microvesicular steatosis. In this sense, prickly pear vinegar treatment helped to reduce the liver affection, although some inflammatory cell infiltration and moderate fibrosis were still observed. The authors suggested that reducing lipid peroxides could be the mechanism underlying the hepatoprotective effect of prickly pear vinegar.

A model of high-fat feeding was also used by Kang et al. [21], who analysed the effects of an extract of *Opuntia ficus-indica* seeds named DWJ504, on non-alcoholic steatohepatitis (NASH). Collected in South Korea, DWJ504 was obtained from the seeds of dried fruits of *Opuntia ficus-indica*, which contained taxifolin (dihydroquercetin), narcissin, isoamericanol A and dihydrokaempferol. For this purpose, male C57BL/6 mice were fed either a normal-fat diet or an HF diet for ten weeks. Over the last four weeks of this dietary treatment, HF-fed mice were distributed into four groups that orally received 0, 250, 500 or 1000 mg of DWJ504/kg b.w./day. The results showed increased hepatic triglycerides and elevated activities of serum ALT and AST in HF mice, which were attenuated by DWJ504 at any dose, without a dose–response pattern. The rest of the parameters were analysed in the mice receiving 500 mg/kg b.w./day. Histopathologic alterations, including inflammatory cell infiltration and necrotic lesions, were reduced. When the authors assessed the potential mechanisms of action underlying these effects, they observed that the increases in the protein expression of sterol regulatory element-binding protein-1 (SREBP-1) and carbohydrate-responsive element-binding protein (ChREBP), two transcription factors involved in *de novo* lipogenesis, were now decreased by the treatment, but not to the levels of mice fed with the normal diet. Regarding β-oxidation, CPT-1, the enzyme that allows long-chain fatty acid to enter the mitochondria to be oxidised, protein expression was increased, although no statistical significance was achieved. In addition, DWJ504 boosted PPAR-α protein expression in the liver. These results indicate that DWJ504 was able to lessen hepatic triglyceride accumulation by reducing fatty acid synthesis and inducing fatty acid oxidation. DWJ504 also increased hepatic-reduced glutathione levels (GSH) and exhibited an anti-inflammatory effect, as shown by the reduction induced in the protein expression of Toll-like receptor-4 (TLR4), nuclear factor-kappa β (NF-kβ), tumour necrosis factor-α (TNF-α) and TIR-domain-containing adapter–inducing interferon β (TRIF) and the gene expression of *Tnf-α*, interleukin 6 (*Il-6*) and interferon β (*Ifn-β*). Moreover, inducible nitric oxide synthase (*iNos*) and Cd40 mRNA levels, markers of pro-inflammatory M1 macrophages, were decreased by DWJ504, whereas arginase 1 (*Arg1*) and macrophage mannose receptor C type 1 (*Mrc1*), markers of anti-inflammatory M2 macrophages, and mRNA levels were increased.

In other studies, diets rich in fat and sucrose were used to induce hepatic alterations. In this line, Sánchez-Tapia et al. [22] investigated the beneficial effects of nopal cladodes (from Mexico) consumption on obese rats fed with a sucrose-enriched high-fat diet, by analysing gut microbiota modifications. For this purpose, male Wistar rats were initially divided into two groups for seven months: the control group and the high fat-sucrose group (HFS), the latter receiving a diet with 45% kcal from fat and 5% sucrose added to the drinking water. After this period, HFS rats were divided into four groups and received the following treatments for a month: high-fat-sucrose feeding (HFS group), control feeding (HFS-C group), high-fat-sucrose feeding and 5% dehydrated nopal cladodes (HFS + N group) or control feeding and 5% dehydrated nopal cladodes (HFS-C + N).

One of the most relevant changes observed in gut microbiota was the increase in *Bacteroides fragilis* species in nopal-consuming groups. The circulating levels of lipopolysaccharide (LPS) were also measured, and the results showed a decrease after nopal supplementation. Regarding liver damage markers, the results demonstrated that the groups supplemented with nopal displayed lower expression of the hepatic genes involved in *de novo* lipogenesis, such as *Srebp-1*, fatty acid synthase (*Fas*) and acetyl CoA carboxylase (ACC). Nopal intake also induced a boost in *Ppar-α* mRNA and *Cpt-1*. In addition, the HFS group presented more hepatic inflammation and lipid droplet accumulation in the liver, compared with the other groups. The authors discussed that the observed beneficial effects of *Opuntia* may be due to gut microbiota modifications. Specifically, steatosis reduction could be caused by decreased translocation of LPS, which is associated with an increment in *Bacteroides fragilis*.

More recently, in the study carried out by Héliès-Toussaint et al. [23], the authors fed male rats a standard diet or an HF diet, supplemented or not with cladodes powder from *Opuntia ficus-indica* (collected in Mexico) (0.5% *w*/*w*), for 60 days. The HF diet increased hepatic triglyceride levels, which were slightly, but not significantly, reduced by *Opuntia ficus-indica* treatment. On the other hand, no differences in serum ALT, AST, monocyte chemoattractant protein-1 (MCP-1) and C-reactive protein (CRP) levels were observed.

In summary, all the reported studies addressed in animals showing either genetic steatosis or steatosis induced by high-fat feeding (rats or mice) have shown positive effects of *Opuntia ficus-indica* products on steatosis prevention. The vast majority of the studies have resorted to cladode extracts, although some authors have used either fruit vinegar or seed oil. The effectiveness of the products has been proven with different experimental period lengths, from 4–10 weeks to longer periods of seven months. Concerning the doses, in some cases, the extracts were added to the diet, and, thus, the amount provided to the animals was expressed as a percentage of weight into the diet (0.5, 4 or 5%); in other cases, each animal received a specific amount based on its weight, and, thus, the dose was expressed as mg/kg b.w./day (250, 500 or 1000 mg/kg b.w./day). It should be pointed out that in these studies, the bioactive compound composition has not been provided. Among the main mechanisms of action proposed to explain the anti-steatotic effects of the *Opuntia ficus-indica* products, a reduction in *de novo* lipogenesis, together with an increase in fatty acid oxidation have been deemed important. These changes lead to a reduction in fatty acid availability for triglyceride assembly. Moreover, some studies have observed an improvement in insulin signalling, which is critical since insulin resistance is one of the main causes of liver steatosis. On the other hand, oxidative stress, one of the first insults involved in the development of fatty liver, was reduced by *Opuntia ficus-indica* products. Finally, these compounds also reduce liver inflammation, a process involved in the evolution of liver steatosis towards more advanced stages of non-alcoholic fatty liver disease (NAFLD), such as steatohepatitis.

## 4. Effects of *Opuntia ficus-indica* on Liver Damage Induced by Chemicals

The effects of *Opuntia ficus-indica* have also been studied in models of liver damage induced by different types of chemicals (Table 2). In this line, Galati et al. [24] analysed the effects of the acute or chronic administration of *Opuntia ficus-indica* fruit juice in a model of liver toxicity induced by the intraperitoneal (i.p.) administration of carbon tetrachloride (CCl_4_). This chemical undergoes biotransformation by hepatic microsomal cytochrome P450, leading to the formation of trichloromethyl free radicals that can react with proteins and lipids in the membrane of cell organelles, thus inducing hepatocyte necrosis. *Opuntia ficus-indica* was collected in Sicily. In the acute treatment, two hours after CCl_4_ administration, rats were distributed into three experimental groups: group I with no further treatment, group II treated with *Opuntia ficus-indica* fruit juice and group III treated with 0.1 g silymarin/kg b.w., used as a protective reference drug. At 24 h, 48 h and 72 h after CCl_4_ administration, six animals from each group were euthanised. Rats in group I showed increased serum ALT and AST in comparison to the controls, the maximum levels observed taking place 24 h after CCl_4_ administration. Compared to group I, group II presented reduced values for both markers at 24 h or 48 h after CCl_4_ administration. Similar results were found in group III. Histological analysis showed migrating macrophages and some damage around the centrilobular vein with necrotic cells, as well as the increased deposition of bundle fibres of collagen, compared with the controls. Acute treatment with *Opuntia ficus-indica* fruit juice (group II) led to a reduction in both degenerative lipidic droplets around the centrilobular vein and vacuolated cells, compared with group I, although inflammatory cells were still present. To address the chronicity experiment, the authors used an additional group treated with *Opuntia ficus-indica* fruit juice for nine days (group IV). These rats displayed a significant reduction in ALT and AST levels, compared with group I. The histological results were similar to those found in the acute treatment, but in this case, an additional decrease in inflammatory cells was observed.

Other authors have induced liver damage by using an insecticide. Thus, Ncibi et al. [25] studied the effect of extracts obtained from *Opuntia ficus-indica* cladodes to ameliorate the damage caused by the insecticide chlorpyrifos (CPF). For this purpose, male mice were fed a chow diet without the administration of any compound (G1) or given 10 mg/kg b.w. of CPF (G2). Other groups of mice received 10 mg/kg b.w. of CPF and 100 mg/kg b.w. of the cactus extract (G3); 150 mg/kg b.w. of CPF (G4); 150 mg/kg b.w. of CPF and 1.5 g/kg b.w. of the cactus extract (G5) or only 1.5 g/kg b.w. of the cactus extract (G6 group), for 48 h. The results demonstrated that half of the mice from G4 (highest dose of insecticide) died after treatment. Furthermore, the surviving mice decreased body weight and increased liver weight, but when they received CPF together with cactus extract, these changes did not occur. Regarding liver function, CPF treatment (G2 and G4) significantly increased blood levels of ALT, AST, lactate dehydrogenase (LDH) and ALP activities, compared to G1. In G3, mice recovered the control values, and among G5, the recovery was partial. Concerning cholesterol, CPF administration significantly increased this parameter, compared with the control group. Mice in G3 and G5 showed no significant differences in cholesterol levels, compared with the control group. It is noteworthy that the administration of the cactus extract alone (G6) did not affect any of the parameters examined.

In another study, the authors analysed the beneficial effects of the juice from *Opuntia ficus-indica* cladodes against the hepatic toxicity caused by nickel chloride (NiCl_2_), which produces hepatotoxicity through oxidative stress promotion and the induction of DNA damage. In this study, Wistar rats were divided into a control group and a group treated with *Opuntia ficus-indica* cladode juice (25% in drinking water) for one month. Rats from each group were then divided into two subgroups, rats i.p. being injected or not with NiCl_2_ daily for 10 days. The authors reported that NiCl_2_ induced a sharp increase in LDH, ALT and AST. Regarding the oxidative status, the hepatotoxic compound induced MDA accumulation and SOD up-regulation, whereas a reduction in both GPx and CAT was observed, prompting greater levels of superoxide anion. *Opuntia ficus-indica* cladode juice was able to completely restore control levels of transaminases and antioxidant enzymes, as well as the lipid peroxidation status [26].

Zourgui et al. [27] studied the effect of an extract of *Opuntia ficus-indica* cladodes (CCE), collected in Tunisia, in Balb/C mice treated with zearalenone (ZEN), a fusariotoxin produced mainly by *Fusarium* species that grows on foodstuffs. Mice were divided into six experimental groups: group 1 (control group) treated with ethanol/water, group 2 treated with 100 mg CCE/kg b.w., group 3 treated with ZEN (40 mg/kg b.w.) and three additional groups treated with doses of 25, 50 or 100 mg/kg b.w. of CCE 24 h prior to 40 mg/kg b.w. of ZEN. Regarding lipid peroxidation, hepatic MDA levels were significantly higher in group 3 than in the controls. The administration of CCE led to a reduction in MDA concentrations and at the lowest dose, control values were recovered. Compared to group 1, protein carbonyl formation was increased in group 3, and the increment was more moderate with previous CCE administration in a dose-dependent manner. Enhanced CAT activity induced by ZEN administration was also observed, an effect that was prevented by the pre-treatment with CCE, also in a dose-dependent manner. Moreover, the authors analysed the effect on the cellular cytoprotective proteins known as heat shock proteins (Hsp), another early marker of oxidative injury. In fact, several studies have reported that many sources of oxidative stress can lead to the up-regulation of *Hsp70* and *Hsp27*. In the present study, the administration of ZEN also promoted the expression of *Hsp70* and *Hsp27* genes in the liver, which had been prevented by the *Opuntia* extract in a dose-dependent manner.

The same group studied the effect of *Opuntia ficus-indica* against the cytotoxicity and genotoxicity of zearalenone [28]. To do so, blab/c mice were distributed in the following experimental groups: group 0, that received water; group 1, that received ethanol/water (1:1, *v:v*); group 2, that received cactus cladode extract at 100 mg/kg b.w.; group 3, that received zearalenone in ethanol/water (1:1, *v:v*) at 40 mg/kg b.w., corresponding to 8% of the LD50; group 4, that received 24 h prior to zearalenone (40 mg/kg b.w.) 25 mg/kg b.w of cactus cladodes extract; group 5, that received 50 mg/kg b.w of cactus cladode extract 24 h prior to zearalenone administration; and group 6, that received 24 h prior to zearalenone (40 mg/kg b.w.) 100 mg/kg b.w of cactus cladode extract. Twenty-four hours after the treatments, the mice were sacrificed. It was observed that zearalenone induced DNA fragmentation in hepatocytes. However, this effect was partially avoided by the cactus cladode extract in a dose-dependent manner, and in the case of the highest dose (100 mg/kg b.w.), it was almost totally avoided. This result is evidence that this extract may neutralize the genotoxic effect of zearalenone, present as a contaminant in food.

Brahmi et al. evaluated the hepatoprotective effect of *Opuntia ficus-indica* cladode extract on aflatoxin B1 (AFB1)-induced hepatic damage [29]. BalbC male mice were distributed in the following groups: group 1, that received water; group 2, that received DMSO/water (1:1, *v:v*); group 3, that received 50 mg/Kg b.w of the extract; group 4, that received AFB1 250 μg/Kg b.w for 15 days; group 5, that received AFB1 250 μg/kg b.w + extract 50 mg/kg b.w (before 15 days treatment by AFB1); group 6, that received AFB1 250 μg/kg b.w + extract 50 mg/kg b.w (after 15 days treatment by AFB1); group 7, that received AFB1 250 μg/kg b.w for 30 days treatment; group 8, given AFB1 250 μg/kg b.w + extract 50 mg/kg b.w (before 30 days treatment by AFB1); and group 9, that received AFB1 250 μg/kg b.w + extract 50 mg/kg b.w (after 30 days treatment by AFB1). *Opuntia ficus-indica* cladode extract at 50 mg/kg b.w. decreased lipid peroxidation when administered before and after AFB1, which was observed by the decrease in the hepatic MDA level. Protein oxidation, determined by protein carbonyl formation, induced by AFB1, was reduced (approximately 60%) by the extract. Regarding heat shock protein expression, the induction of both HSP27 and HSP70 proteins was completely restored by the treatments. These results indicate a reduction in liver damage. Moreover, cladode extract also decreased AFB1-induced genotoxicity, as determined by DNA fragmentation, a chromosome aberrations test and an SOS Chromotest. As far as apoptosis is concerned, it should be noticed that the protein expression of p53 and BAX was increased by the cladode extract, whereas BCL2 was significantly decreased. These results pointed out a pro-apoptotic effect of the extract.

It is well known that a good method to induce liver injury is chronic ethanol administration. This was the model used by Alimi et al. [30] to address the effects of *Opuntia ficus-indica f. inermis* prickly pear juice, recollected in Tunisia. Thus, the authors induced hepatotoxicity in Wistar rats by the chronic administration of ethanol (3 g/kg b.w.) through intragastric intubation for 90 days. The results showed that ethanol caused a sharp increase in AST, ALT, ALP, LDH and gamma-glutamyl transferase (GGT) levels. Regarding oxidative stress, chronic ethanol ingestion down-regulated SOD, CAT, GPx enzymes and reduced glutathione (GSH) levels, whereas both lipid and protein oxidation were raised. Interestingly, *Opuntia ficus-indica f. inermis* juice was able to partially restore these modifications to those of the control levels, at the dose of 20 mL/kg b.w. In addition, the highest dose of prickly pear juice (40 mL/kg b.w.) completely prevented the alterations prompted by ethanol-induced hepatotoxicity. The histopathological analysis revealed that the administration of the prickly pear juice improved the fatty vacuoles produced by the chronic ethanol intake.

Saad et al. [31] promoted liver damage by administering lithium. The authors used an extract obtained from the cladodes of *Opuntia ficus-indica*, collected in Tunisia. This extract contained 125.01 ± 0.90 mg of phenolic compounds, expressed as gallic acid equivalents (GAE), and 71.02 ± 0.76 mg of flavonoids, expressed as quercetin equivalents (QE). The identified phenolic acids were gallic acid, catechin, caffeic acid, epicatechin, vanillic acid and coumarin, and two other unknown compounds. In the case of flavonoids, seven compounds were found, four of which were known, namely, rutin, isorhamnetin, quercetin and kaempferol. Male Wistar rats were divided into four experimental groups: the control group, a group treated with lithium carbonate (25 mg/kg b.w./day, i.p. administered) to induce oxidative stress, a group treated with *Opuntia ficus-indica* extract (100 mg/kg b.w.) for 60 days and distilled water for the last 30 days of treatment, and a group which received *Opuntia ficus-indica* extract for 60 days and lithium carbonate for the last 30 days. Lithium carbonate administration induced increased values of serum transaminases (ALT, AST, ALP and LDH), which were attenuated in rats treated with the *Opuntia ficus-indica* extract. Moreover, this extract prevented the lithium-induced increase in the MDA level, as well as the decrease observed in SOD, CAT and GPx, evidencing its powerful protective effect on oxidative lithium-induced damage in rats.

Tahri-Joutey et al. [32] aimed to evaluate the hepatoprotective effect of cactus seed oil (CSO) from *Opuntia ficus-indica* against LPS-induced damage. For this purpose, mice were fed on a control diet supplemented or not with 6% (*w*/*w*) CSO for 28 days. Four hours before euthanasia and during the fed state, half of the animals of each group received an injection (5 mg/kg b.w.) of 100 µg of LPS. These groups were named control (+LPS) and CSO (CSO + LPS). Regarding inflammation markers, LPS administration significantly increased hepatic interleukin-1β (*Il-1β*) mRNA levels and IL-1β protein. In the case of oxidative stress, both *Sod1* mRNA expression and CAT activity were augmented in the liver of LPS-treated mice. Furthermore, AOX enzymatic activity was reduced with LPS treatment. Concerning the beneficial effects of CSO, although inducible nitric oxide synthase (iNOS) protein expression was not affected by any treatment, CSO significantly reduced LPS-induced interleukin-10 (*Il-10*) mRNA expression. Moreover, CSO was able to attenuate the increase in LPS-induced *Sod1* mRNA. In the case of AOX activity, its value was restored almost to control levels. In conclusion, CSO showed hepatoprotective effects against short-term LPS-induced liver metabolic stress due to its capacity to repair the peroxisomal antioxidant and fatty acid β-oxidation functions.

To summarise, different chemicals have been used to induce liver damage in the gathered studies: carbon tetrachloride, chlorpyriphos insecticide, nickel chloride, fusariotoxin zearalenone, ethanol, lithium and lipopolysaccharide. In all cases, *Opuntia ficus-indica* products were useful in reversing or preventing (depending on the experimental design used) the liver damage induced by these compounds. No clear conclusion can be drawn concerning the doses because a great variety exists in the studies in terms of the units used: (a) 3–40 mL of juice, (b) 25–1500 mg of extract/kg body weight or (c) 25% in the diet. The beneficial effects observed consisted of triglyceride accumulation, oxidative stress and/or inflammation. The positive effects on oxidative stress were mainly due to an increase in SOD, CAT and GPx activities as well as in GSH. Concerning the effects on inflammation, the beneficial effects were induced via Il-1β, Il-6 and Il-10.

## 5. Effects of Other *Opuntia* Species on Models of Non-Alcoholic Liver Disease

In addition to *Opuntia ficus-indica*, the effects of other *Opuntia* species on liver steatosis and different stages of NAFLD have also been analysed (Table 3). Héliès-Toussaint et al. [23] fed male rats a standard diet or an HF diet, supplemented or not with cladodes powder of *Opuntia streptacantha*, collected in Mexico (0.5% *w*/*w*), for 60 days. The HF diet increased hepatic triglyceride concentration, and this effect was not prevented by F-OSC treatment. With regard to the liver and inflammation, no differences in serum ALT, AST, MCP-1 and CRP levels were shown between the experimental groups.

Chahdoura et al. [33] analysed the effect of *Opuntia microdasys* on liver steatosis in a model of type 2 diabetes. For this purpose, *Opuntia microdasys* flowers at the post-flowering stage were collected in Tunisia and used to prepare an aqueous extract. Rats were distributed into seven groups: healthy rats (C), healthy rats treated with 100 mg of *Opuntia microdasys* decoction/kg b.w./day (F3C1), healthy rats treated with 200 mg of *Opuntia microdasys* decoction/kg b.w./day (F3C2), diabetic rats (C+), diabetic rats treated with 100 mg metformin/kg b.w./day (Met), diabetic rats treated with 100 mg of *Opuntia microdasys* decoction/kg b.w./day (D + F3C1) and diabetic rats treated with 200 mg of *Opuntia microdasys* decoction/kg b.w./day (D + F3C2). The treatment lasted four weeks. Type 2 diabetes was induced by administering a fructose solution (30%) to rats for two weeks prior to treatment, and an injection of 60 mg alloxan/kg b.w. 24 h before the treatment. The authors observed increased liver weight in diabetic rats, compared with the healthy group. The ingestion of *Opuntia microdasys* in diabetic rats seemed to moderate the boost. Hepatic ALT, AST, ALP and total bilirubin were higher in the diabetic rats than in the control group. When diabetic rats ingested an *Opuntia microdasys* decoction (D + F3C1 and D + F3C2 groups), ALT was reduced. Similar results were found with the treatment of metformin, a commonly used anti-diabetic drug. Concerning oxidative stress, MDA and protein carbonylation were increased in diabetic rats, while D + F3C2 and Met treatments decreased these markers. SOD, CAT and GPx activities were also reduced in the diabetic rats and their activity was restored, particularly with D + F3C2 treatment, probably due to the reactive oxygen species (ROS) scavenging ability of *Opuntia microdasys*. Whereas the control F3C1 and F3C2 rats showed normal liver tissue and hepatocyte structure, the diabetic rats presented liver damage, characterised by mononuclear cell infiltrations, the enlargement of the portal vein and necrotic cells with reduced and condensed nuclei. The Met and D + F3C2 groups revealed an improvement in the histopathological analysis, showing normal hepatocytes, sinusoids and central vein and the absence of leukocyte infiltration and necrotic cells.

Lastly, Zouaoui et al. [34] studied the protective effects of *Opuntia dejecta* flowers (from Tunisia) against liver alterations induced by type 2 diabetes in rats. Thus, Wistar rats were fed or not with a high-fructose (10% fructose) diet for 20 weeks, and two further groups were supplemented orally with flower extract of *Opuntia dejecta* (100 and 300 mg/kg b.w./day) over the last 4 weeks. In 100 g, the extract contained 1503 mg of phenolic compounds, 809 mg of flavonoids, 753 mg of flavonols, 141 mg of tannins and 122 mg of ortho-benzenediol. The authors observed that, compared to the control group, rats under a high-fructose diet presented high levels of AST, ALT transaminase and ALP, and their livers showed necrosis, fibrosis, lymphocytic infiltration and steatosis features concurrently. Furthermore, SOD, CAT and GPx antioxidant activities were increased in diabetic rats, which prompted a rise in lipid peroxidation. On the contrary, when the animals were supplemented with an extract of *Opuntia dejecta*, this partially prevented the increase induced by the high-fructose diet in both transaminases and MDA accumulation. In addition, the antioxidant/oxidant imbalance displayed in diabetic rats was completely prevented, at least with the highest dose of *Opuntia dejecta* extract.

In summation, three studies have analysed the effects of products obtained from different species of *Opuntia*, at doses of 100–300 mg/kg body weight, or at 0.5% in the diet, on the alterations observed in the liver from animal models exhibiting steatosis induced by high-fat feeding or steatosis associated with type 2 diabetes. In the case of *Opuntia streptacantha*, no beneficial effects were observed on liver triglyceride accumulation, serum transaminase levels or inflammation. Regarding *Opuntia microdasys* and *Opuntia dejecta*, they both reduced serum ALT levels and oxidative stress, although, in these studies, the triglyceride amount in the liver was not measured. In the case of *Opuntia streptacantha*, it is not possible to know whether the extract obtained from the cladodes was indeed ineffective, or whether the lack of effect observed was due to some other factors of the experimental design, such as the dose or the length of the experimental period.

## 6. Effects of Other *Opuntia* Species on Liver Damage Induced by Chemicals

As in the case of *Opuntia ficus-indica*, the effects of other *Opuntia* species have also been studied in models of liver damage induced by different types of chemicals (Table 4). Saoudi et al. [35] investigated the protective properties of an *Opuntia vulgaris* extract, collected in Tunisia, in a model of methanol-induced biochemical and oxidative damage. The total phenolic content of the extracts was 107 ± 1.3 mg GAE/g and the total flavonoids were 18.7 ± 0.5 mg, expressed as QE/g. Adult male albino Wistar rats were divided into two experimental groups: control rats (C) and rats treated with *Opuntia vulgaris* fruit aqueous extract (OE; given as a beverage) for six weeks. After this period, each group was divided into two subgroups; one of them was injected daily with 2.37 g methanol/kg b.w. for four weeks in order to induce liver damage. ALT, AST, ALP, LDH and bilirubin were augmented in the methanol-injected group in comparison with the control group, but the administration of OE reversed these effects. SOD, CAT and GPx activities in the liver were reduced with methanol treatment and levels were restored with OE. Methanol also induced increased hepatic lipid peroxidation, although OE prevented this effect. After treatment with methanol, liver tissue and cell structures were damaged, and the OE pre-treatment also averted this deleterious effect. The authors proposed that the OE properties may be due to the superoxide-radical-scavenging activity, likely associated with the antioxidant compounds of *Opuntia vulgaris*.

González-Ponce et al. [36] analysed the hepatoprotective effect of *Opuntia robusta* and *Opuntia streptacantha* fruits, collected in Mexico, on acetaminophen (APAP)-induced liver damage. Male Wistar rats were divided into the control group (C), a group treated with a single dose of APAP (500 mg/kg b.w., i.p.; APAP), a group treated with 800 mg *Opuntia robusta*/kg b.w./day (*Or*), a group treated with 800 mg *Opuntia streptacantha*/kg b.w./day (*Os*), a group treated with *Opuntia robusta* followed by a single dose of APAP (*Or* + APAP), a group treated with *Opuntia streptacantha* with a subsequent single dose of APAP (*Os* + APAP) and a group pretreated with 50 mg GSH/kg b.w./day with a posterior single dose of APAP (GSH + APAP). The composition of the extracts was as follows: *Opuntia robusta* extract contained 574 ng/L of phenolic compounds, 89 μg of flavonoids/mL, 329 mg of ascorbic acid/L, 467 mg of betalains/L, 333 mg of betacyanin/L and 134 mg of betaxanthin/L. *Opuntia streptacantha robusta* extract contained 343 ng/L of phenolic compounds, 54 μg of flavonoids/mL, 66 mg of ascorbic acid/L, 124 mg of betalains/L, 187 mg of betacyanin/L and 36 mg of betaxanthin/L. It is noteworthy that the administration of GSH and both *Opuntia* species counteract the depletion of GSH induced by APAP. The experimental period in all the cacti extract treatments lasted five days. The single dose of APAP led to the generation of the highly-reactive metabolite N-acetyl-p-benzoquinone-imine (NAPQI), which resulted in GSH depletion and the subsequent reaction of NAPQI with cellular proteins and lipids to form APAP adducts, which damage mitochondria. The results showed that APAP treatment increased serum transaminases and ALP, but the prophylactic administration of both *Opuntia robusta* (*Or* + APAP group) and *Opuntia streptacantha* (*Os* + APAP group) significantly decreased the values of all the liver cell injury markers more efficiently than GSH administration (GSH + APAP group). The extracts alone produced no changes.

In addition, APAP treatment caused an array of liver cell damage, consisting of extensive hydropic vacuolation, glycogen depletion and the focal necrosis of cells with pyknotic nuclei, compared to the control group. The liver injury was prevented in the groups *Or* + APAP, *Os* + APAP and GSH + APAP. The authors suggested that the antioxidant effect of *Opuntia* extracts may be due to their components. Moreover, both species demonstrated free radical scavenging capacity in vitro, although this capacity was higher in the case of the *Opuntia robusta* extract. Nonetheless, *Opuntia streptacantha* had a higher chelating activity. In vitro analysis also showed that *Opuntia* extracts reduced the APAP-induced toxicity in liver cells more than N-acetylcysteine (NAC).

Four years later, the same authors published an article [37] where Wistar rats were treated or not with a single dose of APAP (500 mg/kg b.w., i.p.), and the same cactus extracts (800 mg/kg b.w.) were orally administered in a single dose after 30 min. In this case, after six hours of hepatic intoxication, the rise induced in transaminases and LDH levels by APAP was partially prevented by both extracts. Similarly, GSH depletion and APAP-induced lipid peroxidation were partially and completely restored, respectively, by *Opuntia robusta* and *Opuntia streptacantha* extracts. It is noteworthy that cactus extracts also avoided the negative effects prompted in antioxidant-related genes (*Sod2*, *superoxide dismutase 2*; *Gclc*, *glutamate-cysteine ligase* and *Hmox1*, *heme oxygenase 1*) and in the stress-related gene *Gadd45b* (*growth arrest and DNA-damage-inducible*). With regard to the histopathological analysis, *Opuntia* extracts lessened either the ballooning or necrosis of APAP-induced hepatocytes. Surprisingly, *Opuntia robusta* seemed more protective than *Opuntia streptacantha* extract against the liver degeneration prompted by APAP, probably because *Opuntia robusta* extract presented a significantly higher concentration of betacyanins (2.21 fold).

Recently, researchers of the same group (Villa-Jaimes et al. (2022) [38]) aimed to investigate whether the same cactus extracts (800 mg/kg b.w.) were able to protect against diclofenac-induced liver injury. For this purpose, Wistar rats were divided into a control group, a diclofenac-treated group (DF; 75 mg/kg b.w., i.p., one dose), an *Opuntia robusta* + diclofenac (OR + DF) group or an *Opuntia streptacantha* + diclofenac (OS + DF) group, being rats pre-treated with a daily oral dose of OR or OS (800 mg/kg b.w./day) for five days prior to liver damage induction by diclofenac. As it was expected, diclofenac significantly increased serum AST and ALT levels after liver damage induction; this rise in transaminase levels was prevented by the prophylactic administration of both *Opuntia* extracts. It is important to mention that the preventive effect exerted by OS pre-treatment was greater in comparison with that of the OR extract. Regarding liver hematoxylin-eosin staining, livers from the DF group showed marked vacuolation and cell necrosis. Nevertheless, these features of liver damage were prevented by OR or OS pre-treatment extracts. To study the mechanism of action of both extracts more in depth, these authors also carried out an in vitro study with primary rat hepatocytes incubated for 12 or 24 h with diclofenac (400 μmol/L), with or without OR/OS extracts, added 30 min after diclofenac exposure. Therefore, both OR and OS extracts were able to prevent the mitochondrial ROS production (measured by MitoSOX probe) induced by diclofenac exposure. In the same line, the co-incubation with the *Opuntia* extracts totally avoids the up-regulation of caspase-3 induced by diclofenac-induced liver injury.

Zhu X et al. [39] analysed the effect of the phenolic extract isolated from *Opuntia stricta* cladode juice, from Tunisia, in cadmium- (Cd-)-induced hepatic damage. Therefore, Wistar rats were divided into the following groups: rats receiving just normal saline (G1); rats given cladode juice extract (250 mg/kg b.w./day) (G2); rats administered cadmium chloride (CdCl_2_) (1 mg/kg b.w./day) (G3); and rats fed with CdCl_2_ (1 mg/kg b.w./day) together with cladode juice extract (G4) for five weeks. The *Opuntia stricta* included in their chemical composition phenolic and flavonoid contents of 24.71 ± 3.93 mg GAE/g dry weight and 8.84 ± 0.41 QE/g extract, respectively. The results demonstrated that rats treated with CdCl_2_ had significantly higher levels of ALT, AST and bilirubin in the liver, compared to the controls. G4 showed significantly lower levels of these parameters, compared to G3. Regarding enzymatic antioxidants, rats receiving just CdCl_2_ reduced hepatic SOD, CAT and GPx, but these decreases were prevented when treated with cladode juice extract. Moreover, both lipid peroxidation and CdCl_2_-induced protein carbonylation levels were lower when rats received cladode juice extract. These parameters were also lower in G2, compared to G1. CdCl_2_ administration also increased a key hepatic marker of liver disease, metallothionein (MT), but the levels were only significantly reduced when they were treated with cladode juice extract. G3 showed the highest Cd concentration in the liver, and levels were significantly lower in rats from G4 (CdCl_2_ + *Opuntia stricta* extract). The histopathological analysis showed that CdCl_2_ produced focal hepatocyte swelling, vacuolation and inflammation, whereas cladode juice extract administration preserved the morphology and restored the architectures of this organ.

*Opuntia dillenii* is another species that has been studied in the last few years by several authors. Bouhrim et al. [40] analysed the protective effect of *Opuntia dillenii* seed oil (ODSO) from Morocco on the hepatotoxicity induced by CCl_4_. Wistar rats were divided into the following experimental groups: the control group received 10 mL distilled water/kg b.w./day, a second group received CCl_4_ intraperitoneally (1 mL/kg body weight) once a week for two weeks of treatment, and a third group received ODSO (2 mL/kg) once daily, for one week until the first intraperitoneal injection of CCl4, and then ODSO administration for 7 days until the second injection of CCl4. Regarding liver damage, CCl_4_ showed higher liver weight, and increased serum biomarkers, such as ALT, AST, ALP, bilirubin, TG and uric acid. However, plasma glucose levels were diminished after CCl_4_ administration in comparison with the control group. It is noteworthy that all these parameters of liver injury were partially prevented by the pre-treatment with ODSO. With regard to oxidative status, hepatic MDA increased in the CCl_4_ group, and ODSO was able to prevent lipid peroxidation in the liver. In addition to this study, the same authors (Bouhrim et al. [41]) also analysed the beneficial effects of the same extract of *Opuntia dillenii* seed oil on liver injury induced by gentamicin. Wistar rats were divided into the following experimental groups: the control group received 10 mL distilled water/kg b.w./day (NCG), a second group received water before an injection of 80 mg gentamicin/kg b.w./day, i.p. (GCG), and a third group received 2 mL ODSO/kg b.w./day orally, before gentamicin administration (OOG) for two weeks. Regarding liver damage, GCG showed higher serum GGT levels that were attenuated with ODSO pre-treatment (OOG group). There were no significant differences between groups in the serum ALP level. With regard to oxidative status, hepatic MDA increased with gentamicin administration and ODSO pre-treatment decreased this parameter.

Shirazinia et al. [42] investigated the hepatoprotective effect of an *Opuntia dillenii* fruit hydroalcoholic extract (OHAE), collected in Iran, on liver toxicity, oxidative stress and inflammation induced by lead acetate, the most common environmental contaminants in the Earth’s crust. The total phenolic content was 65 mg GAE/g dried extract. The experimental time was ten days, divided into two periods of five days each. For this purpose, Wistar rats were divided into the following experimental groups: the control group received distilled water orally during the first and second five-day periods, and physiological saline (0.9% *w*/*v* NaCl, i.p.) daily during the second five-day phase; the Pb group was treated with lead acetate and received distilled water during the first and second periods and lead acetate (25 mg/kg b.w./day, i.p.) during the second phase; the OHAE100 group received OHAE (100 mg/kg b.w./day) during the first and second periods and lead acetate during the second phase, and the OHAE200 group received OHAE (200 mg/kg b.w./day) during the first and second periods, and lead acetate during the second phase. Regarding hepatic markers, ALT, AST and ALP were increased in the Pb group, compared to the control group. OHAE-treated rats showed lower values when compared with the Pb group. In addition, lead acetate also reduced serum CAT activity and augmented MDA levels, which were reversed by OHAE treatment at both doses. The histopathological analysis showed that although the Pb group developed liver damage, this was improved with both OHAE doses. Pathological scores were higher in the Pb group, and they were significantly reduced in the OHAE200 group.

Pulido-Hornedo et al. [43] analysed the antioxidant, hepatoprotective and antifibrotic effects of *Opuntia robusta* (*Or*) from Mexico on rats exhibiting hepatic fibrosis, one of the stages developed in NAFLD and caused by thioacetamide (TAA). For this purpose, TAA was administered twice a week, the first time at a dose of 250 mg/kg b.w. and the second time at a dose of 200 mg/kg b.w. Male Wistar rats were divided into the following groups: control, TAA, *Opuntia robusta* pulp extract (*OrP*; 800 mg/kg b.w./day), *Opuntia robusta* extract (*OrE*; 800 mg/kg b.w./day), betanin (25 mg/kg b.w./day), *OrP*/TAA, *OrE*/TAA, betanin/TAA and NAC/TAA. The treatment lasted five weeks, and samples of blood and liver tissue were taken on the 2nd, 3rd, 4th and 5th weeks to analyse the effect of the treatments during the different fibrosis stages. The *OrP* chemical composition was 436 ± 57 mg betacyanin equivalents/L for betacyanins, 1118 mg GAE/100 g dry matter basis (dmb) for total phenols and 793 mg catechin equivalents (CAE)/100 g dmb for flavonoids.

The results showed that TAA administration augmented serum ALT and AST levels. Although in the 3rd week AST levels were not significantly different between *OrP*/TAA and the control group, the *OrE*/TAA and NAC/TAA groups showed a significant reduction in the 4th week of treatment, compared with the TAA group. In the last week, all treatments reduced AST levels, compared to TAA, and the administration of *OrP* and *OrE* to rats treated with TAA also decreased ALT levels significantly, compared to the TAA group. Moreover, this group showed lower hepatic GSH levels than the control rats, but the betanin/TAA and both *Or*/TAA groups demonstrated a higher GSH content than the controls, especially in the 4th week of treatment. During the 2nd and the 3rd weeks, *OrE*/TAA treatment showed the highest GSH levels, and in the 4th week, the *OrP*/TAA group had the highest concentrations. On week 5, all the rats receiving TAA showed a significantly lower GSH concentration, compared to the control group. This oxidative stress prompted by TAA caused a boost in MDA levels, but they were significantly decreased with *OrE*/TAA treatment at weeks 2, 3 and 4, being more effective than betanin/TAA treatment at weeks 3 and 4. The histopathological analysis demonstrated that TAA generated extensive areas of vacuolation, necrosis and fibrosis, inflammatory infiltration and a loss of the normal architecture of the liver parenchyma. Both *Or* treatments significantly reduced the liver damage caused by TAA administration, although the whole fruit extract seemed more effective than the pulp extract.

**Table 4:** In vivo studies analysing the effect of extracts obtained from different *Opuntia* species on liver damage induced by chemicals.

Reference	*Opuntia* Species	Cacti Part	Animal Model	Experimental Design	Effects	Mechanisms
Saoudi et al. (2012) [35]	*Opuntia vulgaris*	Fruit aqueous extract	Male Wistar rats	Chemical product: 2.37 g methanol/kg b.w., i.p., for 4 weeks more Pre-treatment: drinking *Opuntia* fruit extract for 6 weeks Experimental period: 10 weeks	↓ Cell structure ↓ Serum ALT, AST, ALP, LDH and bilirubin ↓ Oxidative stress ↓ Lipid oxidation	↓ Oxidative stress: ↑ SOD, CAT and GPx activities
González-Ponce et al. (2016) [36]	*Opuntia robusta* and *Opuntia streptacantha*	Fruit extracts	Male Wistar rats	Chemical product: 500 mg APAP/kg b.w., i.p. Pre-treatment: 800 mg *O. robusta* or 800 mg *O. streptacantha*/kg b.w./day, for 5 days Experimental period: 6 days	↓ Hydrophic vacuolation, focal necrosis of cells ↓ Serum ALT, AST and ALP ↓ Oxidative stress	↓ Oxidative stress: ↑ GSH levels
González-Ponce et al. (2020) [37]	*Opuntia robusta* and *Opuntia streptacantha*	Fruit extracts	Male Wistar rats	Chemical product: 500 mg APAP/kg b.w., i.p. Treatment: 800 mg *O. robusta* or 800 mg *O. streptacantha*/kg b.w./day Experimental period: 1 day	↓ Ballooning, necrosis ↓ Serum ALT, AST, ALP and LDH ↓ Oxidative stress	Oxidative stress: ↑ GSH levels ↓ MDA ↑ *Sod2*, *Gclc*, *Hmox1* and *Gadd45b* mRNA
Pulido- Hornedo et al. (2022) [43]	*Opuntia robusta*	Pulp and extract	Male Wistar rats	Chemical product: Twice a week 250 mg TAA/kg b.w., i.p. and 200 mg TAA/kg b.w., i.p. Treatment: 800 mg *O. robusta* pulp or 800 mg *O. robusta* extract/kg b.w./day Experimental period: 5 weeks	↓ Serum ALT and AST ↓ Oxidative stress	↓ Oxidative stress: ↑ GSH levels ↓ MDA (with *O. robusta* extract treatment)
Villa-Jaimes et al. (2022) [38]	*Opuntia robusta* and *Opuntia streptacantha*	Fruit extracts	Male Wistar rats	Chemical product: 75 mg diclofenac/kg b.w., i.p. Treatment: 800 mg *O. robusta* or 800 mg *O. streptacantha*/kg b.w./day Experimental period: 5 day	↓ Necrosis ↓ Serum ALT, AST ↓ Oxidative stress and apoptosis (in vitro)	Oxidative stress: ↓ Mitochondrial ROS (in vitro) Apoptosis: ↓ Caspase-3 activity (in vitro)
Zhu et al. (2022) [39]	*Opuntia stricta*	Cladode juice extract	Male Wistar rats	Chemical product: 1 mg CdCl_2_/kg b.w./day, i.p. Treatment: 250 mg cladode juice extract/kg b.w./day Experimental period: 5 weeks	↓ Focal hepatocyte swelling, vacuolation and inflammation ↓ Serum ALT, AST, bilirubin and MT ↓ Hepatic Cd ↓ Oxidative stress	↓ Oxidative stress: ↑ SOD, CAT and GPx activities ↓ MDA and protein carbonylation
Bouhrim et al. (2018) [40]	*Opuntia dillenii*	Seed Oil (ODSO)	Wistar rats	Chemical product: 1 mL CCl_4_/kg b.w./once a week, i.p. Treatment: 2 mL ODSO/kg b.w./day Experimental period: 2 weeks	↓ Serum ALT, AST, ALP, bilirubin, TG and uric acid ↓ Oxidative stress	↓ Oxidative stress: ↓ MDA
Bouhrim et al. (2021) [41]	*Opuntia dillenii*	Seed oil (ODSO)	Wistar rats	Chemical product: 80 mg gentamicin/kg b.w./day, i.p. Treatment: 2 mL ODSO/kg b.w./day Experimental period: 2 weeks	↓ Serum GGT ↓ Oxidative stress	↓ Oxidative stress: ↓ MDA
Shirazinia et al. (2021) [42]	*Opuntia dillenii*	Fruit hydroalcoholic extract (OHAE)	Male Wistar rats	Chemical product: 25 mg lead acetate/kg b.w./day, i.p. for last 5 days Pre-treatment: 100 mg OHAE or 200 mg OHAE/kg b.w./day for 10 days Experimental period: 10 days	↓ Histopathologic damage ↓ Serum ALT, AST and ALP ↓ Oxidative stress	↓ Pathological scores (just with 200 mg OHAE/kg b.w./day dose) ↓ Oxidative stress: ↑ CAT activity ↓ MDA

ALP: alkaline phosphatase, ALT: alanine aminotransferase, APAP: acetaminophen, AST: aspartate aminotransferase, b.w.: body weight, CAT: catalase, Cd: cadmium, CdCl_2_: cadmium chloride, GGT: gamma-glutamyl transferase, *Gadd45b*: growth arrest and DNA-damage-inducible, *Gclc*: glutamate-cysteine ligase, GPx: glutathione peroxidase, GSH: reduced glutathione, *Hmox1*: heme oxygenase 1, HF: high fructose, HF: high-fat diet, i.p.: intraperitoneally, LDH: lactate dehydrogenase, MDA: malondialdehyde, MT: metallothionein, ODSO: *Opuntia dillenii* seed oil; OHAE: *Opuntia* hydroalcoholic extract; ROS: reactive oxygen species; SOD: superoxide dismutase, TAA: thioacetamide, TG: triglyceride. The ↑ and the ↓ arrows represent the processes augmented and suppressed by *Opuntia* spp., respectively

In summary, the published studies focused on how different species of *Opuntia* show that products (extracts or juice) obtained from *Opuntia vulgaris*, *Opuntia robusta*, *Opuntia streptacantha*, *Opuntia microdasys*, *Opuntia dillenii* and *Opuntia dejecta* were able, at doses of 100–250 mg/kg body weight, or 2 mL/kg body weight, to attenuate liver damage induced by several chemicals, such as methanol, acetaminophen, cadmium, gentamicin, lead acetate or thioacetamide. As in the case of *Opuntia ficus-indica*, the main beneficial effect on liver damage is the reduction in oxidative stress. In general terms, the reported *Opuntia* products increased SOD, CAT and GPx activities as well as the level of GSH.

## 7. Concluding Remarks

Data reported in the literature and gathered in the present review show that there is scientific evidence supporting the beneficial effects of extracts and other derived products of several *Opuntia* species on the alterations induced in the liver as a consequence of the genetic background, an inadequate feeding pattern or the administration of chemical products.

Concerning the studies addressing the alterations induced by inadequate feeding patterns, all of them studied the preventive effects of *Opuntia* products because they were administered alongside the experimental diets. Consequently, further studies are needed to analyse the potential correction of liver alterations when they are already established. Among all the experimental designs used in the reported studies, those based on inadequate feeding patterns or ethanol administration are the most representative of human pathologies. Nevertheless, knowing the effect that *Opuntia* products have on the alterations induced in the liver by the administration of chemicals provides valuable information regarding the potential of these products in liver health.

It is important to emphasize that in the vast majority of the studies, a profile of bioactive compounds present in the *Opuntia* products has not been provided. This is an important limitation because to reproduce the effects of plant extracts, it is essential to know their composition and standardise their production. On the other hand, the lack of information concerning the bioactive compound profile does not allow for the association of the therapeutic effects of these plants to the presence of specific compounds in the extracts or to know if the observed effects result from additive or even synergistic effects of these compounds.

**Figure 2:**
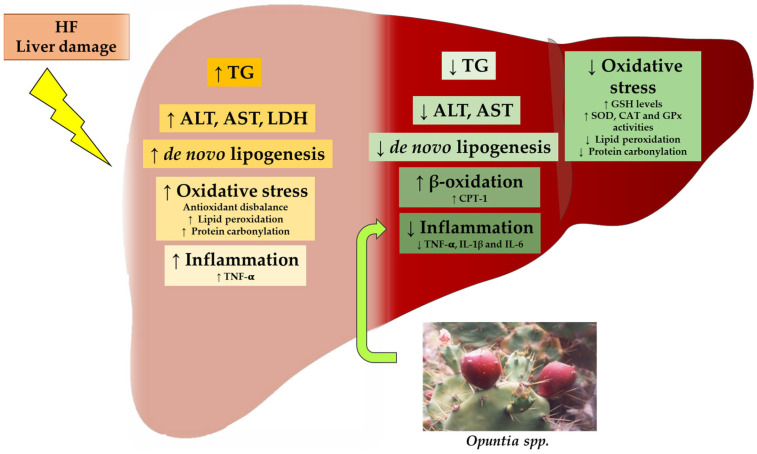
Graphical summary of potential mechanisms by which *Opuntia* spp. modulate lipid accumulation, oxidative stress and inflammation associated with fatty liver. ALT: alanine aminotransferase, AST: aspartate aminotransferase, CAT: catalase, CPT-1: carnitine palmitoyltransferase 1, GPx: glutathione peroxidase, GSH: reduced glutathione, HF: high-fat diet, IL-1β: interleukin 1β, IL-6: interleukin 6, LDH: lactate dehydrogenase, SOD: superoxide dismutase, TG: triglyceride, TNF-α: tumour necrosis factor α. The ↑ and the ↓ arrows represent the processes augmented and suppressed by *Opuntia* spp., respectively.

In some studies, the authors have described some of the mechanisms of action potentially involved in the beneficial effects observed. In general terms, the reduction in triglyceride accumulation can be attributed, in part, to the increase in fatty acid oxidation and the reduction in *de novo* lipogenesis, which leads to a decreased availability to synthesize triglycerides. In the case of steatosis and liver damage induced by chemicals, a strong impact that leads to a reduction in oxidative stress is observed. *Opuntia* products also show interesting anti-inflammatory effects (Figure 2).

The results published to date are promising, but further studies are required to gain more insight concerning (1) the bioactive compound profiles associated with positive effects on the liver, (2) the dose–response patterns and (3) the additional mechanisms of action underlying the beneficial effects, among others. Moreover, to use *Opuntia* products as nutraceuticals in the prevention and/or management of liver alterations, it is necessary to verify whether the positive effects observed in animal models are also found in human beings.

## Figures and Tables

**Table 1:** In vivo studies analysing the effect of *Opuntia ficus-indica* on liver steatosis.

Reference	Cactus Part	Animal Model	Experimental Design	Effects	Mechanisms
Morán-Ramos et al. (2012) [19]	Cladode	Male Zucker (*fa*/*fa*) rats	Diet: standard Treatment: 4% of dietary fibre from nopal in place of cellulose Experimental period: 7 weeks	↓ Hepatic lipid content ↓ Serum ALT, AST and MDA ↓ Serum insulin	↓ Hepatic lipid content: ↑ β oxidation: ↑ *Ppar-α*, *Cpt-1* and *Aox* gene expression ↑ CPT-1 protein expression ↑ Insulin signalling: ↑ IRS1 and AKT phosphorylation
Bouazza et al. (2015) [20]	Fruit vinegar	Male Wistar rats	Diet: control or HF Treatment: 7 mL prickly pear vinegar/kg b.w./day Experimental period: 7 months	↓ Hepatic lipid content ↓ Inflammatory cells ↓ Serum AST ↓ Oxidative stress	↓ Oxidative stress: ↑ SOD and GPx activities ↑ Iron and copper levels
Kang et al. (2016) [21]	DWJ504 seed extract	Male C57BL/6 mice	Diet: control or HF (for the last 4 weeks) Treatment: 250, 500 or 1000 mg/kg b.w./day Experimental period: 10 weeks	↓ Hepatic lipid content ↓ Serum ALT, AST ↓ Inflammation ↓ Necrotic lesions ↓ Oxidative stress (↑ hepatic GSH)	↓ Hepatic lipid content: ↓ Fatty acid synthesis (↓ SREBP-1, ChREBP and PPAR-γ protein expression) ↑ β oxidation (↑ PPAR-α protein expression) ↓ Inflammation: ↓ TLR4, NF-Κβ, TNF-α and TRIF protein expression ↓ *Tnf*-α, *Il-6* and *Ifn-β* mRNA ↓ *iNos* and *Cd40* mRNA ↑ *Arg1* and *Mrc1* mRNA ↓ Oxidative stress: ↑ Hepatic GSH)
Sanchez-Tapia et al. (2017) [22]	Nopal cladodes	Male Wistar rats	Diet: control or HFS Treatment: 5% dehydrated nopal Experimental period: 7 months	↓ Hepatic lipid content ↓ Hepatic inflammation ↓ Fasting serum glucose levels	↓ Hepatic lipid content: ↓ *De novo* lipogenesis (↓ *Srebp-1*, *Fas* only in rats fed with the HFS diet) and *Acc* mRNA) ↑ β oxidation (↑ *Cpt*-*1* and *Ppar-α* mRNA (only in rats fed with the HFS diet) ↓ Mucose permeability (↓ serum LPS and gut occludin) Changes in gut microbiota
Héliès-Toussaint et al. (2020) [23]	Cladode powder	Male Sprague- Dawley rats	Diet: HF Treatment: powder 0.5% *w*/*w* Experimental period: 7 months	No significant beneficial effects	

ACC: acetyl-CoA carboxylase, AKT: protein kinase B, ALT: alanine aminotransferase, AOX: acyl-CoA oxidase 1, *Arg1*: arginase 1, AST: aspartate aminotransferase, b.w.: body weight, CPT-1: carnitine palmitoyltransferase-1, *Fas*: fatty acid synthase, GPx: glutathione peroxidase, GSH: reduced glutathione, HF: high-fat, HFS: high-fat sucrose, *Ifn-β*: interferon β, *Il-6*: interleukin 6, *iNOS*: inducible nitric oxide synthase, IRS1: insulin receptor substrate 1, LPS: lipopolysaccharide, MDA: malondialdehyde, *Mrc1*: macrophage mannose receptor, C type 1, NF-Kβ: nuclear factor-kappa β, PPAR-α: peroxisome proliferator-activated receptor-α, SOD: superoxide dismutase, SREBP-1: sterol regulatory element-binding protein-1, TLR4: Toll-like receptor-4, TNF-α: tumour necrosis factor-α, TRIF: TIR-domain-containing adapter–inducing interferon β. The ↑ and the ↓ arrows represent the processes augmented and suppressed by *Opuntia* spp., respectively.

**Table 2:** In vivo studies analysing the effect of *Opuntia ficus-indica* on liver damage induced by chemicals.

Reference	Cactus Part	Animal Model	Experimental Design	Effects	Mechanisms
Galati et al. (2005) [24]	Fruit juice	Male Wistar rats	Chemical product: 1 mL CCl_4_/kg b.w., i.p. Treatment: 3 mL *Opuntia* fruit juice or 0.1 g silymarin/kg b.w. Experimental period: 24 h, 48 h, 72 h or 9 days	↓ Hepatic lipid content ↓ Inflammatory cells (after 9 days of treatment) ↓ Serum ALT and AST	
Ncibi et al. (2007) [25]	Cladode extract	Male SWISS mice	Chemical product: 10 mg insecticide chlorpyrifos (CPF)/kg b.w. or 150 mg CPF/kg b.w. Treatment: 100 mg *Opuntia* extract/kg b.w. or 1.5 g *Opuntia* extract/kg b.w., respectively Experimental period: 2 days	↓ Serum ALT, AST, ALP, LDH and TC	
Hfaiedh et al. (2008) [26]	Cladode juice	Male Wistar rats	Chemical product: 4 mg (30 µmol) NiCl_2_/kg b.w., i.p. for 10 days Pre-treatment: 25% cladode juice in drinking water Experimental period: 1 month	↓ Serum ALT, AST and LDH ↓ Oxidative stress	↓ Oxidative stress: ↓ MDA and SOD ↑ GPx and CAT
Zourghi et al. (2008) [27]	Cladode extract (CCE)	Balb/C mice	Chemical product: 40 mg ZEN/kg b.w., i.p. Pre-treatment: 25 mg CCE, 50 mg CCE or 100 mg CCE/kg b.w. Experimental period: 24 h	↓ Oxidative stress	↓ Oxidative stress: ↓ MDA, protein carbonylation and CAT activity ↓ *Hsp70* and *Hsp27* mRNA
Zourghi et al. (2009) [28]	Cladode extract (CCE)	Balb/C mice	Chemical product: 40 mg ZEN/kg b.w., i.p. Pre-treatment: 25 mg CCE, 50 mg CCE or 100 mg CCE/kg b.w. Experimental period: 24 h	↓ DNA fragmentation	
Brahmi et al. (2011) [29]	Cladode extract (CCE)	Balb/C mice	Chemical product: 250 μg AFB1/kg b.w., i.p. Pre-treatment: 50 mg CCE CCE/kg b.w. (after or before AFB1) Experimental period: 15 or 30 days	↓ Liver damage ↓ Genotoxicity ↑ Apoptosis	↓ Liver damage: ↓ MDA, protein carbonylation ↓ HSP70 and HSP27 proteins ↓ Genotoxicity: ↓DNA fragmentation, chromosome aberrations test and SOS Chromotest ↑ Apoptosis: ↑p53 and BAX proteins ↓ BCL2 protein
Alimi et al. (2012) [30]	Fruit juice	Male Wistar rats	Chemical product: 10 mL ethanol/kg b.w. (1 p.m.) Treatment: 20 mL fruit juice or 40 mL fruit juice/kg b.w. (9 a.m.) Experimental period: 3 months	↓ ALT, AST, ALP, LDH and GGT ↓ Oxidative stress	↓ Oxidative stress: ↑ SOD, CAT and GPx activities ↑ GSH ↓ MDA and protein carbonylation
Saad et al. (2017) [31]	Cladode extract (CCE)	Male Wistar rats	Chemical product: 25 mg Li carbonate/kg b.w., i.p., 2 times/day (during the last month of treatment) Treatment: 100 mg CCE/kg b.w./day Experimental period: 2 months	↓ Serum ALT, AST, ALP and LDH ↓ Oxidative stress ↓ Serum glucose	↓ Oxidative stress: ↑ SOD, CAT and GPx activities ↓ MDA
Tahri-Joutey et al. (2022) [32]	Seed oil	Male C57BL/6J mice	Chemical product: a single dose of 100 µg of LPS, i.p. (5 mg/kg b.w.) 4 h before euthanasia Pre-treatment: control diet + 6% *w*/*w* cactus seed oil Experimental period: 28 days	↓ Inflammation ↓ Oxidative stress	↓ Inflammation: ↓ *Il-1β*, *Il-6* and *Il-10* mRNA (when LPS was administered) ↓Oxidative stress: ↑ *iNos* (when LPS was not administered) mRNA ↑ *Cat* (when LPS was not administered) and *Acox* (when LPS was administered) mRNA ↓ *Sod1* (when LPS was administered) mRNA ↑ CAT activity (when LPS was not administered)

AFB1: aflatoxin B1, ALP: alkaline phosphatase, ALT: alanine aminotransferase, AST: aspartate aminotransferase, b.w.: body weight, CAT: catalase, CCE: cladode extract, CCl_4_: carbon tetrachloride, CPF: chlorpyriphos insecticide, GGT: gamma-glutamyl transferase, GPx: glutathione peroxidase, GSH: reduced glutathione, *Hsp27*: heat shock protein 27, *Hsp70*: heat shock protein 70, *Il-1β*: interleukin-1β, *Il-6*: interleukin 6, *Il-10*: interleukin-10, *iNOS*: inducible nitric oxide synthase, i.p.: intraperitoneally, LDH: lactate dehydrogenase, Li: lithium, LPS: lipopolysaccharide, MDA: malondialdehyde, NiCl_2_: nickel chloride, SOD: superoxide dismutase, TC: total cholesterol, ZEN: zearalenone. The ↑ and the ↓ arrows represent the processes augmented and suppressed by *Opuntia* spp., respectively.

**Table 3:** In vivo studies analysing the effect of extracts obtained from different *Opuntia* species on non-alcoholic fatty liver disease.

Reference	*Opuntia* Species	Cacti Part	Animal Model	Experimental Design	Effects	Mechanisms
Héliès- Toussaint et al. (2020) [23]	*Opuntia streptacantha*	Cladode extract	Male Sprague- Dawley rats	Diet: control or HF Treatment: 0.5% *w*/*w O. streptacantha* extract Experimental period: 2 months	No significant beneficial effects	
Chahdoura et al. (2017) [33]	*Opuntia microdasys* (Lehm)	Aqueous extract of flowers at post-flowering stage (OFP)	Male Wistar rats	Diet: control or HFr Treatment: 100 mg OFP or 200 mg OFP/kg b.w./day Experimental period: 28 days	↓ Serum ALT ↓ Oxidative stress	↓ Oxidative stress: ↑ SOD, CAT and GPx activities (with 200 mg OFP/kg b.w./day) ↓ MDA and protein carbonylation (with 200 mg OFP/kg b.w./day)
Zouaoui et al. (2021) [34]	*Opuntia dejecta*	Extract flowers at post- flowering stage	Male Wistar rats	Diet: control or HFr Treatment: 100 mg OFP or 300 mg OFP/kg b.w./day, during the last month Experimental period: 5 months	↓ Serum ALT, AST and ALP ↓ Oxidative stress	↓ Oxidative stress: ↑ SOD, CAT and GPx activities ↓ MDA accumulation

ALP: alkaline phosphatase, ALT: alanine aminotransferase, AST: aspartate aminotransferase, b.w.: body weight, CAT: catalase, GPx: glutathione peroxidase, HFr: high fructose, HF: high-fat diet, MDA: malondialdehyde, OFP: *Opuntia* extract of flowers at post-flowering stage, SOD: superoxide dismutase. The ↑ and the ↓ arrows represent the processes augmented and suppressed by *Opuntia* spp., respectively
